# Strategies to enhance graphic and results interpretation of a regression-based approach for method comparison studies

**DOI:** 10.4155/fsoa-2017-0004

**Published:** 2017-04-21

**Authors:** Salvatore Sotgia, Arduino A Mangoni, Gianfranco Pintus, Ciriaco Carru, Angelo Zinellu

**Affiliations:** 1 Department of Biomedical Sciences, School of Medicine, University of Sassari, Sassari, Italy; 2 Department of Clinical Pharmacology, School of Medicine, Flinders University and Flinders Medical Centre, Adelaide, Australia; 3 Department of Biomedical Sciences, College of Health Sciences, Qatar University, Doha, Qatar; 4 Quality Control Unit, University Hospital Sassari (AOU), Sassari, Italy

**Keywords:** acceptance limits, agreement, coefficient of determination, correlation, inherent combined imprecision, intercept, logarithmic transformation, method comparison studies, regression, slope, straight regression

## Abstract

To improve the effectiveness of a previous regression-based approach for the assessment of the agreement between different analytical methods, two modifications/integrations to the original scheme by means of log10 transformation of data and implementation of inherent combined imprecision are presented in this study.

## Lay abstract

Despite its inadequacy, a scatter plot combined with correlation and/or regression analysis to assess the agreement between analytical methods has a long tradition in clinical chemistry. To overcome the problems of correlation and/or regression analysis, a general regression-based test was published in 2008. In this article, two further improvements to this approach are proposed.

Despite the existence of a number of statistical approaches for the comparison of an established analytical method with a new candidate assay, their implementation and interpretation remain challenging for many analysts. This is mainly due to their excessive technicality, which greatly undermines applicability for research purposes. In this context, therefore, it is not surprising that using scatter plots, combined with a correlation and regression analysis, has been widely misapplied for the evaluation of the agreement between analytical methods. The popularity of these statistical tools is likely due to their ease to perform and interpret, along with their high graphical impact. Moreover, these approaches are familiar among the analysts because agreement assessment is performed in the same way as when a linear relation between two variables is searched. The critical point, however, is that correlation and least squares linear regression analysis both measure the strength of a linear association, but not the agreement between analytical methods [1]. To overcome the issues linked to the classical regression/correlation method, while retaining its ease of execution and interpretation, a general regression-based test was published by Sotgia *et al*. in 2008 [2]. To further improve the effectiveness of this approach, two modifications to the original procedure are proposed here: the log10 transformation of data to enhance the impact of the XY chart; and the combination of the coefficient of determination (R^2^) with the acceptance limits based on the inherent combined imprecision (ICI) [3], to facilitate decision making on the agreement between the methods under comparison.

## Fallacy of classical regression/correlation analysis in method comparison studies

**Figure 1.**
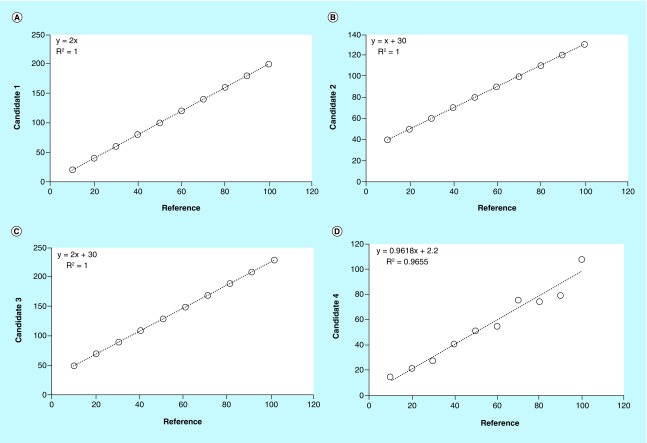
**Comparisons between reference versus candidate methods by ordinary least squares method.** The slope of 2 and intercept of 0, signal a proportional systematic error of 100% between methods **(A)**; the slope of 1 and intercept of 30, signal a constant systematic error of 30 between methods **(B)**; the slope of 2 and intercept of 30, signal both a proportional and constant systematic error of 100% and 30, respectively, between methods **(C)**; the R^2^ below 1 does not allow to evaluate the magnitude of the error between the methods by means of the slope and intercept **(D)**.

To better understand the limits of these approaches in the assessment of agreement between analytical methods, refer to data in Table 1 where a series of hypothetical measurements of the same quantity performed by means of five fictive analytical methods are reported. Candidate methods generate values that are higher than those of the reference method. Using correlation/regression analysis, it would be expected that disagreement between reference versus candidates is clearly signaled both by the coefficients of correlation (r) and of determination (R^2^), and by the least-square parameters (slope and intercept) of the equation of the best fit line. When the agreement between the methods is relatively low, in fact, data points should be scattered around, but not close to, the best fit line, and both r and R^2^ values should be progressively close to 0 depending on the disagreement degree. Similarly, both intercept and slope should differ from those of the equality line, which are, by definition, 0 and 1, respectively. As shown in Figure 1A, contrary to what is expected and despite candidate 1 produces estimate values that are exactly twice those obtained with the reference method, all points lie exactly on the regression line and both r and R^2^ have a value of 1. In other words, r and R^2^ show a perfect linear relationship despite a clear disagreement between the methods under comparison, thus demonstrating their limitations in the correct assessment of agreement. On the other hand, regardless of the real agreement between the measurements, it is sufficient that data are linearly and positively correlated in order to obtain high values of r and R^2^. Although these parameters should not be used to assess the agreement *per se*, they highlight the potential value of using the slope and intercept of the best fit line to evaluate the agreement between the analytical methods. Both slope and intercepts are, in fact, highly sensitive, respectively, to proportional and constant errors. However, they can be used effectively for this purpose only when r and/or R^2^ have a value of 1 [1]. In this situation, in fact, the systematic error in the candidate method is constant within the whole range of the concentrations and, therefore, the slope and intercept signal exactly by how much the data obtained using the candidate method differ from those obtained using the reference 1. Therefore, considering again the scenario in Figure 1A, where r and R^2^ are both 1 and the equation of the best fit line shows a slope of 2 and an intercept of 0, it can be concluded that between reference versus candidate 1 there is a proportional error of 100% without any constant error. By contrast, the comparison between reference versus candidate 2 (Figure 1B) produces a slope of 1 and an intercept of 30, thus revealing the lack of a proportional error and the presence of a constant error of 30. Finally, the comparison between reference versus candidate 3 (Figure 1C) generates a slope of 2 and an intercept of 30, signaling the presence of both a proportional error of 100% and a constant error of 30. Unfortunately, the ability of the slope and the intercept to identify both the type and especially the extent of the errors between two methods, is lost when r and R^2^ values are <1 (Figure 1D). In such a condition, slope and intercept become difficult to interpret and any considerations on the agreement based on their values are speculative.

**Table 1.** **Measurements of a hypothetical analyte performed by five fictive analytical methods.**

**Reference**	**Candidate 1**	**Candidate 2**	**Candidate 3**	**Candidate 4**
10	20	20	30	15
20	40	30	50	22
30	60	40	70	28
40	80	50	90	41
50	100	60	110	51
60	120	70	130	55
70	140	80	150	76
80	160	90	170	75
90	180	100	190	80
100	200	110	210	108

## Sotgia's approach

**Figure 2.**
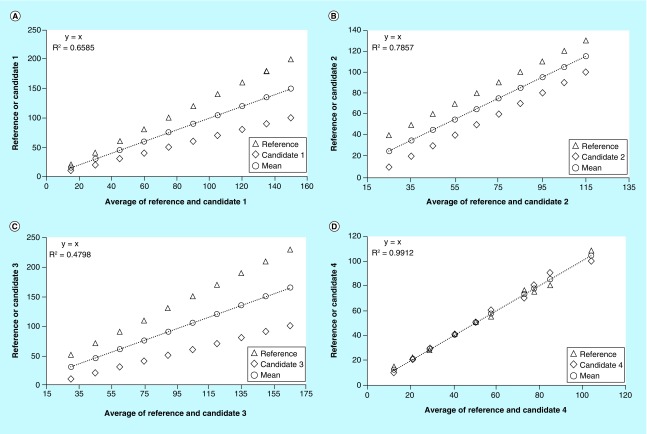
**Comparisons between reference versus candidate methods by the original procedure as it was proposed by Sotgia *et al* [2].** Disagreement between reference vs. candidate 1, candidate 2, and candidate 3 was signaled by mutual distance of estimates, their distance from the best fit line, and by the relatively low R^2^ values of 0.6585 **(A)**, 0.7857 **(B)**, and 0.4798 **(C)**. A substantial agreement between reference versus candidate 4 was instead signaled by a R^2^ of 0.9912, which was above of the cut-off value of 0.98 **(D)**.

In the procedure of Sotgia *et al*., the estimates (Y) obtained by the analytical methods under comparison, both the new and old methods, are plotted against the averages of each pair of the estimate values (X). Using the least square method, the line of the best fit and R^2^ as well as the equation of the straight regression are then computed. The average points of each pair of estimates will lie exactly on the best fit line, and the equation of the latter will invariably be Y = X. The scattering of the estimates around the best fit line, whose magnitude increases with the increase of the distance of the estimates from each other and, therefore, from their average points, allows a graphical representation of the differences between the methods under comparison. An increase in the degree of the scattering will affect the linear fit. This will result in values of R^2^ gradually smaller than 1, indicating an increasing lack of fit. Thus, the goodness of fit signaled by the values of R^2^, indicates also the goodness of agreement between the methods under comparison. Analyzing, therefore, the data in Table 1 using this procedure allows to clearly assess and visualize the disagreement due to the systematic errors between reference versus candidate methods (see Figure 2A, B & C). This is highlighted by the mutual distance of estimates, their distance from the best fit line and the relatively low R^2^ values of 0.6585, 0.7857 and 0.4798 as shown in Figure 2A, B and C, respectively. However, there may be situations where the agreement or disagreement may not be immediately clear as in the previous examples. In Figure 2D, which refers to the comparison of reference versus candidate 4, the scatter of estimates around the regression line is reduced when compared with the previous examples and the value of R^2^ is 0.9912. The original procedure suggests a high agreement between the methods under comparison when R^2^ is equal to or greater than 0.98 [2]. This cut-off was established by several simulations performed by plotting the percentage error between each pair of estimates against their average points, to characterize both the magnitude of the error and its distribution, and by crossing the obtained results with those supplied by other statistical tests. On average, for a cut-off of 0.98, the percentage error between the methods under comparison ranges from 5 to 10%. Thus, in the example above mentioned, a cut-off of 0.98 is able to signal a substantial agreement between reference versus candidate 4 (R^2^ = 0.9912 > 0.98). However, the strict application of this rule can be misleading, especially when R^2^ is close to the cut-off point. In addition, when the measured values are distributed over a wide range, it is also difficult to visualize the mutual distance of the points at low values, for example, the first versus last pair of values in Table 1 in the comparison of reference versus candidate 1 (see Figure 2A).

## Sotgia's approach: modified

**Figure 3.**
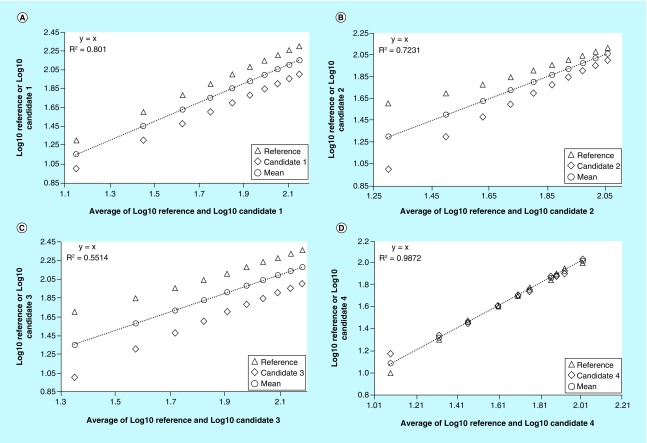
**Comparisons between reference versus candidate methods after log10 transformation of the estimates.**

Taking into account the difficulties that might arise using the original procedure, two modifications are proposed here. The first is to enhance the impact of the XY chart by means of a log10 transformation of data prior to the application of the procedure, which is then executed as described above. It should be noted, however, that the mean of the estimates after their log10 transformation does not correspond to the log10 of their arithmetic mean, but rather to their geometric mean. Moreover, in this context, the log10 transformation is not performed to meet the assumptions of parametric statistical tests, but only to standardize the variations within the distribution of the measured values. Using this approach, the XY graphics in Figure 2 become as shown in Figure 3, where the magnitude of the mutual differences of each pair of values, as well as their distance from the best fit line, is better defined than in Figure 2. The log10 transformation also allows detecting likely outliers which may require further investigation, such as the first pair of estimates in Figure 3D. From the agreement assessment point of view, after the log10 transformation, the R^2^ values change. However, the new values still discriminate the degree of agreement, or lack of, based on a cut-off of 0.98. As shown in Figure 3A, B and C, in the comparison of the reference versus candidate 1, candidate 2 and candidate 3, although R^2^ changes, it remains well below 0.98. Therefore, the disagreement between the methods is confirmed. Similarly, the comparison of reference versus candidate 4, that in the original procedure could be misinterpreted (see Figure 2D), after the log10 transformation further supports the initial interpretation of the agreement between the methods (see Figure 3D). However, the examples discussed so far are based on dummy data and, regardless from the log10 transformation, the sole use of a cut-off of 0.98 can be inadequate to assess the agreement. Therefore, a further proposed change is to integrate the use of R^2^ with the acceptance limits based on the ICI [3], which is an expression of the total imprecisions of the analytical methods under comparison. Total imprecision is related to the random error of the measurements, it has no relation to trueness/accuracy [4], and includes within-day and between-day components of variability [5,6]. It is expressed as %CV or %RSD and it is commonly estimated by means of replicate measurements of different levels of the same samples over multiple days. ICI is computed by the combination of the total imprecisions of the analytical methods that need to be compared, using the following formula:
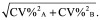



When the analytical methods under comparison (e.g., A vs B) are identical within the ICI, it is expected that 95% of the differences fall within the interval (0 ± 1.96·ICI). Thus, if the total imprecision of a hypothetical method (A) is, for example, 4% and that of another method (B) is 3%, the ICI is
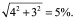



The hypothetical methods A and B are then identical within the ICI, when the 95% of the differences range between 0 ± 1.96·5 = ±9.8%. The implementation of this acceptance criteria in the regression-based test here presented, requires some conceptual adjustments. The procedure, in fact, is not based on the differences or bias between the measurements, but rather on the distances of the estimates from their average points. Therefore, it is expected that analytical methods under comparison are identical within the ICI, when 95% of the estimates fall within the interval (mean ± 1.96·ICI). This means that if the average value of two measurements is 50 and the ICI is, as above, 5%, the estimates are identical within the ICI if they fall within the interval 50 ± 4.9 ([50 ± 1.96·[5/100]·50]). In doing so, an interval around the average value of each pair of estimates can be determined to see how many of them fall in the range encompassed between [mean + 1.96·ICI] and [mean – 1.96·ICI]. Graphically, this is accomplished by drawing the acceptance lines around the best fit line by means of the equations
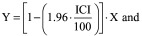


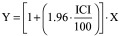



**Figure 4.**
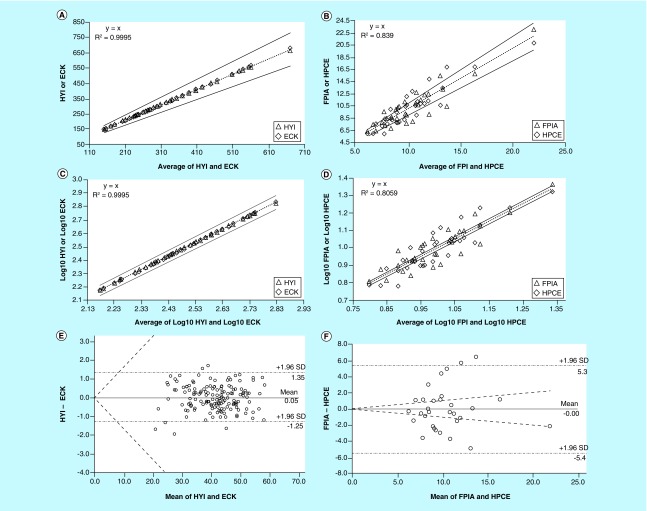
**Comparison of the HYI vs. ECK methods for the measurement of the plasma uric acid concentration by means of the original procedure of Sotgia *et al*. [2].** **(A)** after log10 transformation of the estimates **(C)**, and by Bland-Altman test **(E)**; comparison of FPIA vs. HPCE methods for the measurement of the plasma concentration of the homocysteine by means of the original procedure of Sotgia *et al*. [2]. **(B)** after log10 transformation of the estimates **(D)**, and by Bland-Altman test **(F)**. All of the test were integrated with the limits of agreement defined by the inherent combined imprecision of the methods (solid lines). ECK: Electrokinetic injection; FPIA: Fluorescence polarization immunoassay; HPCE: Capillary electrophoresis; HYI: Hydrodynamic injection.

The log10 transformation of data again improves the graphical output. Obviously, before calculating the acceptance lines, ICI must also be log10 transformed. To better understand the proposed modifications, it is helpful to refer to the Figure 4A, which shows the comparison of real data of plasma uric acid measurements obtained by means of two in-house capillary electrophoresis methods developed by using two different injection modes, hydrodynamic (HYI) versus electrokinetic (ECK). Total imprecisions for HYI and ECK methods were, respectively, 6.6 and 5.1% and the ICI was, therefore,
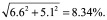



Thus, the limits of acceptance that should contain the 95% of the estimates were mean ± 1.96·ICI = ± 16.35%. As shown in Figure 4A, which illustrates the comparison of HYI versus ECK by means of the original procedure integrated with the acceptance limits based on the ICI, the estimates points mostly lie on the best fit line (dotted line) and the lines of acceptance (solid lines) around the straight regression (dotted line) include more than of the 95% of them, thereby indicating a good agreement within the ICI between the methods. This was further confirmed by the R^2^ value of 0.9995, which was above the cut-off of 0.98. As displayed in Figure 4C, which shows the comparison of HYI versus ECK after log10 transformation, the considerations on the agreement made by means of the R^2^ and acceptance lines, are the same as previously discussed, but the graphical impact of YX chart is significantly improved. The conclusions on the agreement were also confirmed by the Bland–Altman test [7] (Figure 4E). Another example is reported in Figure 4B, D and F which report the comparison of data obtained assaying the human plasma to evaluate the homocysteine concentrations by a fluorescence polarization immunoassay (FPIA) and an in-house capillary electrophoresis method (HPCE). Total imprecision was 2.3% for the FPIA and 4.6% for HPCE, respectively. ICI was therefore
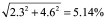



and the limits of acceptance that should contain the 95% of the estimates, were mean ± 1.96·ICI = ±10.07%. Unlike the previous example, in this case the points are widely scattered along the best fit line, both when data are processed by the original procedure (Figure 4B) and after their log10 transformation (Figure 4D), and many of them lie outside the range of acceptance based on ICI. Also in this instance, the XY chart greatly improves after log10 transformation of data (Figure 4D), thus allowing an easier evaluation of the estimates within the range of acceptance. Thus, R^2^ values below the cut-off of 0.98 along with the high number of estimates outside the lines of acceptance, clearly indicate a poor agreement between the FPIA versus HPCE methods. This conclusion was the same as that obtained by Bland–Altman test (Figure 4F).

## Conclusion & future perspective

The arrangement of the bivariate observations in a specific way as described by Sotgia *et al*. [2] yields a least-square line that makes the coefficient of determination sensitive to the systematic errors. The modifications to the original method of Sotgia *et al*. by means of the log10 transformation of data as well as the addition of the limits of the agreement defined by the ICI of the analytical methods under comparison further improve the effectiveness of the procedure in the valuation of the agreement between the analytical methods.

**Executive summary**

- Two changes to the original scheme of the Sotgia's approach for the method comparison studies are proposed.
- The log10 transformation of data prior to the application of the procedure greatly improves the graphical output.
- The use of inherent combined imprecision together with the coefficient of determination (R^2^) facilitates decision making on the agreement between the methods under comparison.
